# Supplementary material for: Immunoengineered magnetic-quantum dot nanobead system for the isolation and detection of circulating tumor cells
Source: J Nanobiotechnology. 2021 Apr 23;19:116. doi: 10.1186/s12951-021-00860-1 (PMC8063296; doi:10.1186/s12951-021-00860-1)
Supplement: Supplementary file 1 — Additional file 1. Characterization of nanoparticles: transmission electron microscopy analysis of FMN; Measurement of hydrodynamic diameter of FMN; Magnetization measurement of FMN; Determination concentration of FMN. Statistical analysis method. Figure S1. Surface xy and Z-stack confocal laser scanning microscopic image of tumor cells captured by iFMNS. Figure S2. Microscope images of control and captured cells for growth of 12 h, 24 h and 36 h. Figure S3. Capture efficiency comparison of FMN and FMN stored at 4 °C for 1 year. Figure S4. Comparison of fluorescent (FL) intensity of quantum dots nanobeads (QDNBs) and FMNs. Table S1. Comparison of iFMNS capturing efficiency at different tumor cell concentration and in various spiking in medium. [file 12951_2021_860_MOESM1_ESM.doc]

**Immunoengineered magnetic-quantum dot nanobead system for the isolation and detection of circulating tumor cells**

Pengfei Zhang,^1,2†^ Mohamed S. Draz,^3,4†^ Anwen Xiong,^5†^ Wannian Yan,^2^ Huanxing Han,^1, 6^* Wansheng Chen^1,7^*

1. Department of Pharmacy, Changzheng Hospital, Second Military Medical University, Shanghai 200003, China.
2. Department of Central Laboratory, Shanghai Skin Disease Hospital, Tongji University School of Medicine, Shanghai 200443, China
3. Department of Chemistry and Chemical Biology, Harvard University, Cambridge, Massachusetts, USA
4. Wyss Institute for Biologically Inspired Engineering, Harvard University, Cambridge, Massachusetts, USA
5. Department of Medical Oncology, Shanghai Pulmonary Hospital, Tongji University Medical School Cancer Institute, Tongji University School of Medicine, Shanghai 200433, China
6. Ailex Technology Group Co., Ltd. Shanghai 201108, China
7. Research and Development Center of Chinese Medicine Resources and Biotechnology, Institute of Chinese Materia Medica, Shanghai University of Traditional Chinese Medicine, Shanghai, 201203, China

^†^ Contributed equally to this manuscript.

*** Correspondence to:**

Prof. Huanxing Han, huanxing_han@163.com

Prof. Wansheng Chen, chenwansheng@smmu.edu.cn

**Characterization of nanoparticles.** For transmission electron microscopy (TEM, JEOL JEM-2100), 2 μl of nanoparticles in ethanol solution were dropped onto a 400-mesh copper grid coated with continuous carbon layer. Size and size distribution of nanoparticles were analyzed with a free software ImageJ. Hydrodynamic diameter of nanoparticles were measured by a dynamic light scattering (DLS) techniques (Zetasizer Nano ZS90, Malvern, UK). Magnetization measurements were performed on a vibrating sample magnetometer (Squid-VSM, Quantum Design, US) at room temperature.

To determine the concentration of FMNs (C_1_) in suspension, the known absorbance (A_1_) of FMNs suspension was weighed after totally drying of centrifugal pellet of FMNs. Then the unknown concentration of FMNs (C_2_) can be calculated from the absorbance (A_2_) according to Lambert-Beer law.

C_2_=C_1_ × A_2_ / A_1_

**Statistical analysis.** We compared differences by one-way ANOVA. We used GraphPad Prism version 6.01 (GraphPad Software) for all statistical analysis. We considered P<0.05 as a statistically significant difference.


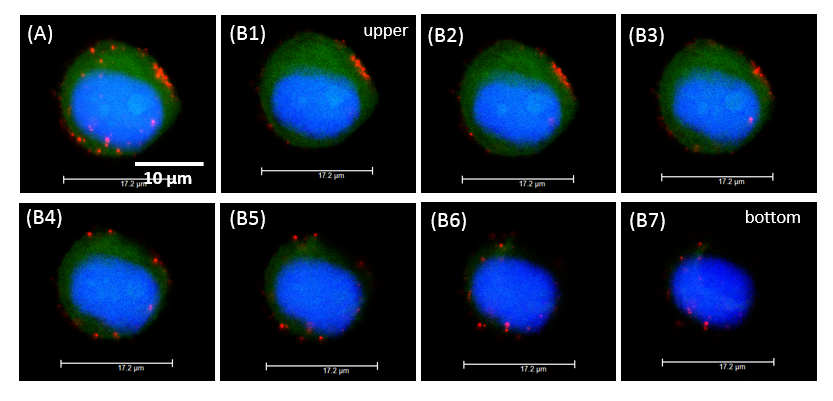


**Figure S1** (A) Surface *xy* confocal laser scanning microscopic image of Celltrace (green) pre-labeled SGC-7901 tumor cells captured by iFMNS (red) and DAPI (blue) stained-cells. (B1-B7) *Z*-stack images obtained at different focalized planes from the upper to bottom of the cell.


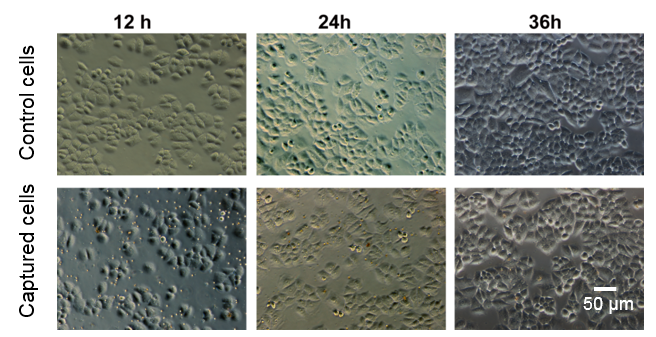


**Figure S2** Microscope images of control and captured cells for growth of 12 h, 24 h and 36 h.


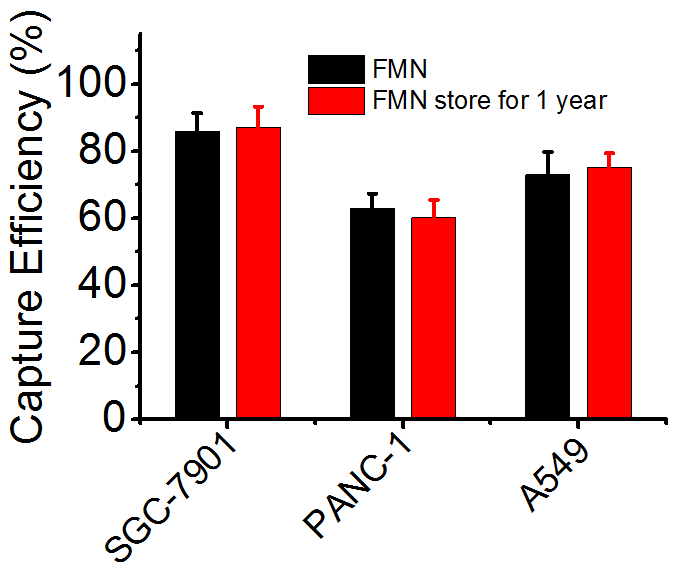


**Figure S3** Capture efficiency comparison of FMN and FMN stored at 4 °C for 1 year.


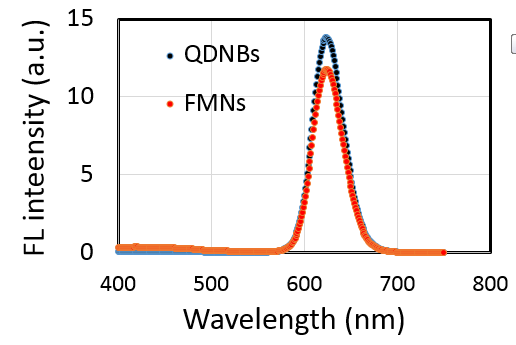


**Figure S4** Comparison of fluorescent (FL) intensity of quantum dots nanobeads (QDNBs) and FMNs.

**Table S1** Comparison of iFMNS capturing efficiency at different tumor cell concentration and in various spiking in medium.

| **NO.** | **1** | **2** | **3** | **4** | **5** |
| --- | --- | --- | --- | --- | --- |
| Number of cells spiking in | 1000 | 500 | 200 | 100 | 50 |
| Captured cells in PBS  (capturing efficiency) | 940  (94%) | 460  (92%) | 192  (96%) | 96  (96%) | 48  (96%) |
| Captured cells in whole blood  (capturing efficiency) | 860  (86%) | 390  (78%) | 140  (70%) | 90  (90%) | 40  (80%) |
| Captured cells in lysis blood  (capturing efficiency) | 867  (86.7%) | 405  (81.0%) | 132  (66%) | 84 (84%) | 45  (90%) |
